# Infection with severe fever with thrombocytopenia virus in healthy population: a cohort study in a high endemic region, China

**DOI:** 10.1186/s40249-021-00918-0

**Published:** 2021-11-16

**Authors:** Xiao-Lei Ye, Ke Dai, Qing-Bin Lu, Yan-Qin Huang, Shou-Ming Lv, Pan-He Zhang, Jia-Chen Li, Hai-Yang Zhang, Zhen-Dong Yang, Ning Cui, Chun Yuan, Kun Liu, Xiao-Ai Zhang, Jiu-Song Zhang, Hao Li, Yang Yang, Li-Qun Fang, Wei Liu

**Affiliations:** 1 State Key Laboratory of Pathogen and Biosecurity, Beijing Institute of Microbiology and Epidemiology, 20 Dong-Da Street, Fengtai District, Beijing, 100071 People’s Republic of China; 2 Department of Laboratorial Science and Technology, School of Public Health, Peking University, Beijing, 100191 People’s Republic of China; 3 Shangcheng Center for Diseases Control and Prevention, Xinyang, 464000 People’s Republic of China; 4 The 990 Hospital of Chinese People’s Liberation Army Joint Logistic Support Force, Xinyang, 464000 People’s Republic of China; 5 Department of Epidemiology, Ministry of Education Key Lab of Hazard Assessment and Control in Special Operational Environment, School of Public Health, Air Force Medical University, Xi’an, 710032 People’s Republic of China; 6 Department of Biostatistics, College of Public Health and Health Professions, and Emerging Pathogens Institute, University of Florida, Gainesville, FL 32611 USA

**Keywords:** Severe fever with thrombocytopenia, Serological study, Healthy participant, IgG antibody, Neutralizing antibody

## Abstract

**Background:**

Severe fever with thrombocytopenia (SFTS) caused by SFTS virus (SFTSV) was a tick-borne hemorrhagic fever that posed significant threat to human health in Eastern Asia. The study was designed to measure the seroprevalence of SFTSV antibody in healthy population residing in a high endemic region.

**Methods:**

A cohort study was performed on healthy residents in Shangcheng County in Xinyang City from April to December in 2018, where the highest SFTS incidence in China was reported. Anti-SFTSV IgG was measured by indirect enzyme-linked immunosorbent assay and neutralizing antibody (NAb) was detected by using PRNT50. The logistic regression models were performed to analyze the variables that were associated with seropositive rates.

**Results:**

Totally 886 individuals were recruited. The baseline seroprevalence that was tested before the epidemic season was 11.9% (70/587) for IgG and 6.8% (40/587) for NAb, which was increased to 13.4% (47/350) and 7.7% (27/350) during the epidemic season, and further to 15.8% (80/508) and 9.8% (50/508) post epidemic. The IgG antibody-based seropositivity was significantly related to the patients aged ≥ 70 years old [adjusted odds ratio (*OR*) = 2.440, 95% confidence interval (*CI*): 1.334–4.461 compared to the group of < 50 years old, *P* = 0.004], recent contact with cats (adjusted *OR* = 2.195, 95% *CI*: 1.261–3.818, *P* = 0.005), and working in tea garden (adjusted *OR* = 1.698, 95% *CI*: 1.002–2.880, *P* = 0.049) by applying multivariate logistic regression model. The NAb based seropositivity was similarly related to the patients aged ≥ 70 years old (adjusted *OR* = 2.691, 95% *CI*: 1.271–5.695 compared to the group of < 50 years old, *P* = 0.010), and recent contact with cats (*OR* = 2.648, 95% *CI*: 1.419–4.941, *P* = 0.002). For a cohort of individuals continually sampled with 1-year apart, the anti-SFTSV IgG were maintained at a stable level, while the NAb level reduced.

**Conclusions:**

Subclinical infection might not provide adequate immunity to protect reinfection of SFTSV, thus highlighting the ongoing threats of SFTS in endemic regions, which called for an imperative need for vaccine development. Identification of risk factors might help to target high-risk population for public health education and vaccination in the future.

**Graphical Abstract:**

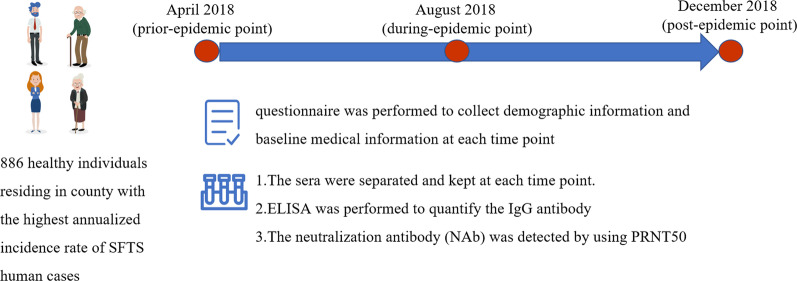

**Supplementary Information:**

The online version contains supplementary material available at 10.1186/s40249-021-00918-0.

## Background

Severe fever with thrombocytopenia (SFTS) is an emerging tick-borne infectious disease caused by SFTS virus (SFTSV), a novel phlebovirus of the Phenuiviridae family, Dabie bandavirus [1]. The disease was firstly reported in China in 2009 and later in other Asian countries in the Republic of Korea, Japan and Vietnam [1–4]. Although typically presenting with mild disease, such as fever, gastrointestinal symptoms, thrombocytopenia and leukocytopenia, SFTSV infection can be life-threatening in 5–30% of the patients [1, 5–7]. The primary infection source is zoonotic, with ticks (mainly *Haemaphysalis longicornis*, *H. longicornis* ticks) acting as the most likely vector for transmitting the virus to humans [7]. The natural reservoir host of SFTSV has not yet been clarified, but it is suggested that various domestic and wild animals in the endemic areas play a role in the maintenance of SFTSV in nature. As has been previously shown, SFTSV antibody was detected in goats, cattle, sheep, pigs, dogs, chicken, rodents, cats, etc. [8–11], posing potential of wide spreading beyond its present enzootic areas via long-distance transfer of animals. China remained as the most severely afflicted country by SFTS, with altogether 7721 confirmed cases reported from 2010 to 2018 [12]. On the other hand, since most of the case finding and investigation efforts had been based on hospital-based surveillance, the mild or subclinical infection might be left unnoticed in the population. Serological studies on the other hand, provided data that could complement traditional symptom-based and laboratory-based surveillance in revealing the infection status among the apparently healthy population or very mild patients who sought no medical care. Under the context of SFTS, the asymptomatic cases had been frequently determined in the SFTS endemic regions, with seroprevalence differing marginally between regions, however, with the limitation of small sample size and inadequate statistical power to explore the risk factors [4, 13–15]. Here we performed a prospective survey on healthy population in the most severely affected region by SFTS disease in China, in order to quantify the risk of SFTSV infection among residents in highly endemic region, to identify risk factors for subclinical SFTSV infection based on large amounts of positive sample and to describe the dynamic change of SFTSV specific antibody level by prospective study design.

## Methods

### Study sites and recruit of participants

From April to December in 2018, a cohort study was performed among healthy populations residing in Shangcheng county in Xinyang City, Henan Province, China (Additional file 1: Fig. S1). Shangcheng county had the highest annualized incidence rate of SFTS human cases (45.95 per 100 000 population) at the county level in Henan Province according to the national reporting data from 2014 to 2016 [16]. Five towns of the county were randomly selected by the cluster sampling method (Wangqiao, Hefengqiao, Wanggang, Guanmiao and Nianyushan towns). From each town, one village was randomly selected as survey sites (i.e., Tumiao, Longtouqiao, Hongfan, Zhaowan and Pingtang villages). For each selected village, all the residents were recruited as the survey participants.

A background investigation using a standard questionnaire was performed on the participants to collect their demographic information (age, gender, local residence duration, occupation, preexisting conditions), and baseline medical information (clinical manifestations that resemble SFTS, the previous diagnosis as SFTS). Those under 18 years old or pregnancy, those who had lived in the area less than 2 years, who had been diagnosed as SFTS or sought medical care due to suspected SFTS, were excluded from the study.

The sample size was estimated by assuming SFTSV-antibody positive rate of 3.0% before epidemic and 6.0% after epidemic, referred from a cross sectional study using a convenience sampling in Xinyang City [17]. Calculated by two-sided binomial test, at least 485 subjects were needed to achieve 90.0% power at a significance level of 0.05, and after taking into account of 10% missing, 534 subjects were needed for the study purpose.

### The prospective observation and sampling of the participants

Three interviews and sampling time points were designed to achieve a consecutive follow-up, which was set in April 2018 (prior-epidemic point); August (during-epidemic point), and December 2018 (post-epidemic point), with comparable intervals. The participants who were sampled during these three time periods were defined as three groups (pre-epidemic samples, during-epidemic and post-epidemic group) for comparison. During each interview effort, a standard questionnaire in Chinese was applied to collect the recent behaviors that was related to SFTS exposure (mostly involving recent tick exposure, field/farming activity, domestic animals feeding and recent exposure to confirmed patients, etc.). The translated questionnaire was provided in the Additional file 1: Table S1.

### Laboratory testing for IgG/IgM antibody and neutralizing antibody

Blood samples were collected from all participants and subject to blood routine test and SFTSV specific antibody test as described as following. The sera were separated and kept at − 80 ℃ until the test in a batch. Briefly, indirect enzyme-linked immunosorbent assay (ELISA) was performed to quantify the IgG antibody titer using commercial kit (Wen Ding BioTech Co., Ltd, Nanjing, China). The sensitivity and specificity of the kit had been tested on 100 serum samples collected within 6 months after recovery of laboratory confirmed SFTS patients, to ensure adequate IgG level to be eligible for positive control. Serum samples collected from healthy people in non-SFTS endemic region in China were used as negative controls. As a result, the total consistency (Kappa value) was estimated to be 0.980, with 98% specificity and 100% sensitivity shown for the ELISA kit. Following the guideline, the tested samples were initially screened at 1:80 dilution, based on which the positive samples were further diluted in twofold from 1:160 to 1:5120 to determine the antibody titer. For each preparation of different dilutions, 100 μl samples and positive/negative/blank control were used, for the measurement of optical density (OD) values at 450 nm. The cutoff value was set as 0.748 × the average OD of negative control + 0.146. If the average OD of negative control was ≤ 0.05, the cutoff value was set as 0.05. A serum sample was considered as SFTSV IgG positive, when a titer of ≥ 1:80 was obtained.

The neutralization antibody (NAb) was detected by using plaque reduction neutralization testing (PRNT50) as previously described [1]. Briefly, the Vero cell-culture of SFTSV strain (WF66) was determined for the infectious dose (CCID50) by microculture cytopathic effect (CPE) assay. The virus storage was tenfold serially diluted with Dulbecco’s modified Eagle medium (DMEM) containing 2% fetal bovine serum and 1% antibiotics, and then 100 μl diluted virus was inoculated into 96-well plate containing 10^5^ Vero per well. After seven days of cultivation at 37 ℃ and 5% CO_2_, CPE was daily observed to calculate CCID50 by the Reed-Muench method. Then the tested serum was twofold serially diluted from 1:20 to 1:640 with DMEM medium containing 2% fetal bovine serum and 1% antibiotics, mixed with the same volume of the diluted virus of 200 CCID50. The same positive and negative control as that for IgG test were simultaneously applied. A titer of ≥ 1:40 was considered as NAb positive. Individuals who were tested positive for either IgG or NAb were considered as having previous subclinical infection, herein referred as subclinical infection for short. Individuals who were tested positive for both IgG and Nab were additionally tested for the presence of anti-SFTSV IgM antibody and SFTSV RNA by real-time RT-PCR.

### Statistical analysis

Based on both IgG and NAb antibody, seropositivity and geometric mean reciprocal titers (GMRTs) were calculated. For those with negative results, half of the detection cut off was used for the calculation of GMRTs. The inter-group difference was estimated by Fisher exact test or non-parametric test where appropriate. The logistic regression models were performed to analyze the variables that were associated with seropositive rates. The variables with *P* < 0.1 in univariate analysis were included in a multivariable logistic regression analysis. Odds ratio (*OR*) and 95% confidence interval (*CI*) were estimated using maximum likelihood methods. All analyses were performed using Stata 14.0 (Stata Corp LP, College Station, TX, USA). A two-sided *P* < 0.05 was considered statistically significant.

## Results

### Seroprevalence and seroconversion

Totally 886 participants were enrolled [median age: 58, interquartile range (IQR): 49–70 years old, 27.1% male]. The median age and gender were largely comparable among three groups, except that the pre-epidemic participants were significantly younger than the other two groups. The frequencies of field activities, including planting vegetables and tea-picking were more frequently reported in during-epidemic survey, while the animal contact was reported with comparable frequencies from three surveys, which reflected the dominant activities that differed across seasons (Additional file 1: Table S2).

Altogether 1445 serum samples (587 pre-epidemic samples, 350 during-epidemic and 508 post-epidemic samples) were tested, with an overall IgG positive rate of 13.6% (197/1445). The seropositive rate increased from 11.9% (70/587, 95% *CI*: 9.4–14.8%) for pre-epidemic samples, to 13.4% (47/350, 95% *CI*: 10.0–17.5%) for during-epidemic samples, and 15.8% (80/508, 95% *CI*: 12.7–19.2%) for post-epidemic samples, with an observable increasing trend although attaining no statistical significance (Chi-square trend test, *P* = 0.183).

The seropositive rate based on NAb was 8.1% (117/1445) as a whole, which increased from 6.8% (40/587, 95% *CI*: 4.9–9.2%) for pre-epidemic samples, to 7.7% (27/350, 95% *CI*: 5.2–11.0%) for during-epidemic samples, and 9.8% (50/508, 95% *CI*: 7.4–12.8%) for post-epidemic samples, again with increasing trend without attaining statistical significance (chi-square trend test,* P* = 0.178).

Altogether 160 individuals with IgG antibody positive results were evaluated for 25 hematological parameters, for whom thrombocytopenia was observed from eight (5.0%) individuals, and leukopenia was observed from six (3.8%) of the individuals, with two of them (1.3%) having both abnormalities. A detailed interview disclosed that none of these individuals had reported recent SFTS like symptoms/syndromes. In comparison with those with normal blood test, these patients had comparable age and gender distribution.

### The contributing factor to anti-SFTSV IgG antibody production

The variables regarding risk behaviors that were obtained at three sampling points were separately analyzed for their association with seropositive results. Univariate analysis on data from before-epidemic season disclosed that 9 out of totally 24 variables achieved *P* < 0.1 for their association with seropositive reaction, among which only one variable (older age ≥ 70 years old) remained significant in the multivariate analysis (adjusted *OR* = 2.430, 95% *CI*: 1.171–5.043, *P* = 0.017). Multivariate analysis for the data from during-epidemic season revealed two factors remained significantly associated with seropositive reaction, including older age ≥ 70 years old (adjusted *OR* = 3.227, 95% *CI*: 1.086–9.588, *P* = 0.035) and close contact with cats in recent two weeks (adjusted *OR* = 2.858, 95% *CI*: 1.179–6.930, *P* = 0.020). For the risk factor analysis at the post-epidemic season, older age ≥ 70 years old remained to be the only significant factor for seropositive reaction (adjusted *OR* = 2.885, 95% *CI*: 1.148–7.254, *P* = 0.024) (Additional file 1: Tables S3, S4).

**Fig. 1:**
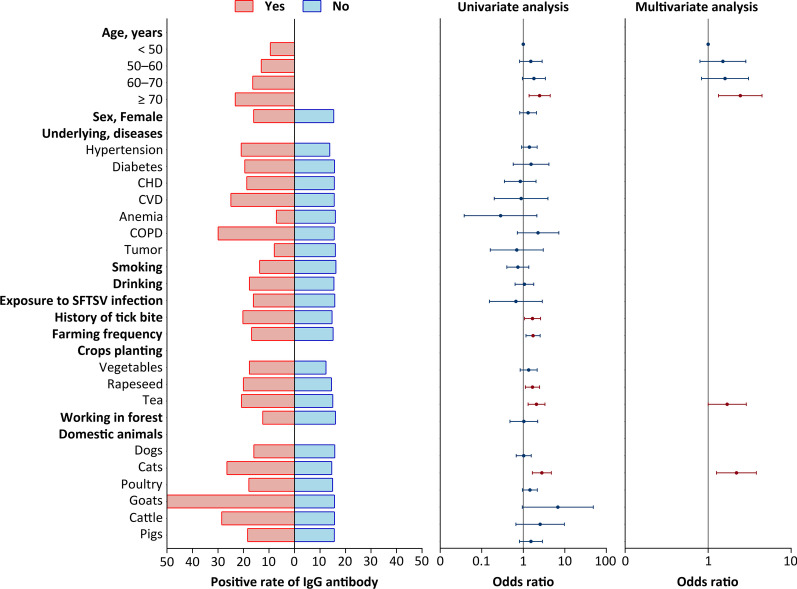
The factors associated with anti-SFTSV IgG seropositive response. All the evaluated factors listed in the left vertical axis were used as categorical variable and the positive rate of sampled individuals within subgroups for each variable were marked by the length of the colored bar (the left column). The odds ratio (*OR*) and 95% confidence interval (*CI*) of the association between evaluated variables and IgG antibody positivity was estimated by the univariate (the middle column) and multivariate (the right column) logistic regression models. CHD, coronary heart disease; CVD, cardiovascular disease; COPD, chronic obstructive pulmonary disease; The variable “Domestic animals” represent contact with the listed domestic animals in recent 2 weeks.

We made a pooled data analysis using three-sampling data from all the 886 participants, by the way that when multiple samples were collected from the same participant, only positive antibody results were used. Among totally 24 variables that were included into the univariate analysis, eight variables attained *P* < 0.1 and were entered into multivariate analysis, from which three variables remained significant, including the patients aged ≥ 70 years old (adjusted *OR* = 2.440, 95% *CI*: 1.334–4.461 compared to the group of < 50 years old, *P* = 0.004), close contact with cats in recent two weeks (adjusted *OR* = 2.195, 95% *CI*: 1.261–3.818, *P* = 0.005), and working in tea garden (adjusted *OR* = 1.698, 95% *CI*: 1.002–2.880, *P* = 0.049) (Fig. 1; Additional file 1: Table S5). We made further age stratified analysis to determine their separate risk factors of acquiring infection. When three age groups were classified, consistent significant results were obtained that included farming frequency in last three months in both < 60 and the 60–70 age groups, planting tea in both 60–70 age and ≥ 70 years old groups. Discrepancy remained for history of tick bite and close contacts with pigs which were only obtained from < 60 age group, for close contacts with cats which was only obtained from the 60–70 age group (Additional file 1: Table S10).

### The contributing factor to anti-SFTSV neutralizing antibody production

**Fig. 2:**
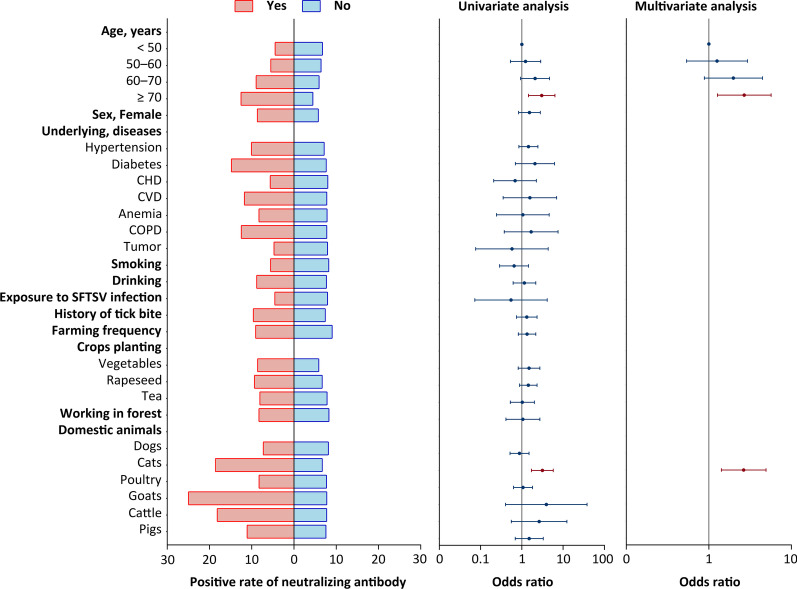
The factors associated with positive anti-SFTSV neutralizing antibody response. All the evaluated factors listed in the left vertical axis were used as categorical variable and the neutralizing antibody positive rate of sampled individuals within subgroups for each variable were marked by the length of the colored bar (the left column). The odds ratio (*OR*) and 95% confidence interval (*CI*) of the association between evaluated variables and neutralizing antibody positivity was estimated by the univariate (the middle column) and multivariate (the right column) logistic regression models. CHD, coronary heart disease; CVD, cardiovascular disease; COPD, chronic obstructive pulmonary disease; The variable “Domestic animals” represent contact with the listed domestic animals in recent 2 weeks.

The same panel of variables was used for the risk factors analysis of anti-SFTSV NAb production as those applied for the IgG antibody analysis. For three-time-point analysis that were separately performed, no significant factor was related to the before-epidemic NAb positivity, while the during-epidemic and post-epidemic NAb positive response was related to close contact with cats in recent two weeks (adjusted *OR* = 5.987, 95% *CI*: 2.318–15.464, *P* < 0.001) and older age ≥ 70 years old (adjusted *OR* = 2.914, 95% *CI*: 1.152–7.370, *P* = 0.001), respectively (Additional file 1: Tables S6, S7). For the combined analysis using all three-time-point data, SFTSV NAb positivity was significantly associated with two variables based on multivariate analysis, i.e., the patients aged ≥ 70 years old (adjusted *OR* = 2.691, 95% *CI*: 1.271–5.695 compared to the group of < 50 years old, *P* = 0.010) and close contact with cats in recent two weeks (*OR* = 2.648, 95% *CI*: 1.419–4.941, *P* = 0.002) (Fig. 2; Additional file 1: Table S8).

### Dynamic pattern of IgG and NAb against SFTSV in the 1-year cohort

A total of 164 participants (median age: 63, IQR: 54–71 years, 32.3% male, both comparable with 950 participants outside of the cohort) had samples collected at all three time points, who were used to profile the dynamic pattern of antibody production during 1-year period. Based on IgG antibody test, the seropositive rate increased from 16.5% (27/164, 95% *CI*: 11.1–23.0%) for before-epidemic season, to 17.1% (28/164, 95% *CI*: 11.7–23.7%) for during-epidemic season, and 18.9% (31/164, 95% *CI*: 13.2–25.7%) for post-epidemic season, without showing significant differences among groups (chi-square trend test, *P* = 0.563). The GMRT of the IgG positive samples was estimated to be 247.55 (95% *CI*: 57.12–1072.9),131.49 (95% *CI*: 5.88–2941.75), and 161.47 (95% *CI*: 6.44–4048.40), respectively for three samplings, without significant trend observed.

Among the 27 participants with positive IgG antibody before epidemic, two (7.4%) turned negative during epidemic, and none turned negative after epidemic. Among the 137 participants with negative IgG antibody before epidemic, three (2.2%) participants turned positive during epidemic, but all turned negative after epidemic, with another six participants turning positive (6/137, 4.4%).

The anti-SFTSV NAb positive rates at three sampling were 6.1% (10/164, 95% *CI*: 3.0–10.9%), 10.4% (17/164, 95% *CI*: 6.2–16.1%), and 10.4% (17/164, 95% *CI*: 6.2–16.1%), respectively (Chi-square trend test, *P* = 0.126). The GMRT of the positive samples for the first sampling was 173.47 (95% *CI*: 14.13–2130), 34.87 (95% *CI*: 0.2–6081.22) and 38.73 (95% *CI*: 0.21–6991.47), respectively, which was similar with the trend displayed for the IgG antibody. For the 10 participants with positive NAb before epidemic, three (30.0%) turned negative during epidemic, and none turned negative after epidemic. Among the 154 individuals who were negative for NAb before epidemic, 10 (6.5%) turned positive during epidemic, with two of them turned negative after epidemic, and another six (4.2%, 6/144) turned positive after epidemic. These seroconverted individuals were same as that for IgG antibody.

### The correlation analysis between IgG and NAb

Among totally 1445 samples that were simultaneously tested for IgG and NAb, 13.6% (197/1445, 95% *CI*: 11.9–15.5%) were IgG positive and 8.1% (117/1445, 95% *CI*: 6.7–9.6%) were NAb positive. 58.9% (116/197) of the positive IgG antibody was related to NAb positive, while 100% (1247/1247) of the negative IgG was related to NAb negative. The agreement between positive detection of IgG and NAb was 80.5% (kappa = 0.709, *P* < 0.001). The higher IgG antibody titer was significantly correlated with NAb positivity (chi-square trend test, *P* < 0.001). For example, within the group with IgG antibody titer of 1:80, the NAb positive rate was 28.9%, which was increased to 54.0% in the group with IgG of 1:160, further to 79.1% in the group with IgG of 1:320 (Additional file 1: Table S9). Totally 117 individuals with positive results for both IgG and Nab were subject to test of SFTSV specific RNA and IgM antibody, from which no positive results were obtained.

## Discussion

The current study showed an overall seropositive rate of 11.9% (IgG antibody) and 6.8% (NAb) among individuals residing in high endemic region. All of those with positive antibodies production denied previous SFTS-like illness, except for hematological abnormalities that were tested from a small proportion of individuals, indicating high prevalence of subclinical or very mild infection. It’s not surprising that the current seroprevalence was higher than most of the previous studies, as our study was performed in the region with the highest endemic level for SFTS, and the case incidence was positively related to the seroprevalence at the population level as previously displayed [17].

On the other hand, the epidemiological risk factors for seropositive reaction indicative of subclinical infection had been investigated, with significance attained for such factors as long-term living in severely epidemic areas [13, 18], living in hilly areas, direct contact with domestic livestock [19], breeding domestic animals (particularly cattle, goats), farming, grazing, tick exposure, etc., all increased the risk of inapparent infection [18–20].The complex enzootic cycles of SFTSV involved interactions between multiple factors, which included competent tick species implicated as vectors, reservoir hosts for the ticks, as well as suitable ecological niche. We hypothesize that the risk factors for SFTSV infection might differ among epidemic seasons, therefore we performed risk factor analysis stratified by epidemic periods, and made comparable and pooled data analysis to corroborate the findings. We determined that older age and close contact with cats were independently related to increased seroprevalence, based on both IgG and NAb antibody evaluation. While only older age was consistently significant across three periods. In agreement with previous findings [17, 21, 22], older individuals were suggested to have increased risk of acquiring infection, possibly due to higher opportunity of exposure to vectors or compromised host immunity. This finding also corresponded with age dependent increase in case incidence rate, suggesting that longer exposure resulted in increased risk of infection, with some of them developing clinical disease.

Behavioral factors for acquiring infection in apparently healthy populations had been previously evaluated, revealing significant effect from raising goats, farming, and grazing activity, however, the conclusion was made only based on 11 seropositive individuals [20]. Here we determined recent contact with cats as the most significant risky behavior that conferred a high risk of acquiring subclinical infection based on the evaluation of both IgG antibody and NAb. The current finding was derived from large size of healthy population, based on data from different epidemic periods, revealing this behavior as consistently related to seropositive response. This finding reinforced previous association that was inferred between owing cats and risk of developing SFTS clinical illnesses [23, 24]. Other previously identified risky factors, such as recent tick bites or presence of tick in the residing or farming area, owing livestock or poultry, farming activity, although were significant contributor to seropositivity in univariate analysis, all had turned into insignificant when performing multivariate analysis except for working in tea garden. Thus, their significant effect from the univariate analysis might be attributable to the higher frequency of risky activity that was more often seen in the older age population, which was adjusted in the current multivariate analysis.

The SFTSV specific antibody had been determined from a high variety of animals, including goats [25, 26], dogs [27] in Korea; wild boars in Japan [28]; from cattle, sheep, goats, deer, and elk in USA[9]; from sheep, cattle, dogs, pigs, and chickens [10]; and wild animals including shrews, rodents [29] and hedgehog [30]. No infection from cat had ever been reported in China [31], this is in contrast with studies in Korea and Japan showing molecular or serological evidence of SFTSV infection from both feral cats and home cats [32, 33], and even SFTS like clinical signs from the infected cats [11]. Experimental evidence also verified their highly susceptible to SFTSV, in that four of the six SFTSV infected cats died, all showing similar or more severe symptoms than human SFTS patients, with high levels of SFTSV RNA detected from serum, eyes, and saliva [34]. A clinical case of cat-to-human transmission of SFTS was recently reported in Japan [35]. Therefore, the current finding reinforced the experiment results or case reports showing that cat might played a more important role in maintaining SFTSV than other domestic animals, such as dog, sheep, pig, poultry or others. Compared with other home-keeping animals, cat might have a higher odd of close contact with human beings, or with higher competence of harboring and transmitting the disease to human beings, therefore acting as a source of direct transmission of SFTSV to humans or other animals. More intensive precautions and education measures are needed for cats, especially among their guardians and veterinarians.

The interpretation of antibody persistence in the healthy population remains challenging. Up to now, most estimation of antibody duration in subclinical individuals were only performed on sporadic cases. In one study in an agricultural population in Jeju Island, Korea, one healthy subject showed seropositivity of IgG antibody over the entire 3-year study period [36]. In another study, eight healthy individuals had neutralizing antibodies detected, and decreased during the subsequent follow up, with three of them turning negative at the 3rd year [37]. Based on the current cohort that was repeatedly sampled, we found comparable proportion of participants who seroconverted from negative to positive or from positive to negative, thus contributing to a largely stable seroprevalence in local residence. The results provided strong evidence supporting that host immunity acquired from natural subclinical infection might not last life long, differing from the long persistent protection elicited from clinical infection in the recovered patients [38]. On the other hand, there was the same individual who underwent marginally increased antibody titer after one epidemic season, which might suggest a host immunity boost through re-exposure to SFTSV although displaying no clinical disease.

The current ELISA testing results was not fully congruent with neutralization assay, despite of high correlation between two measurements. The IgG antibody production might also represent a cross reactivity to other concomitant circulating Bunyavirus due to the similar risk of acquiring SFTSV infection and other bunyaviruses. As we have mentioned in the methods, the ELISA kit used in this study for SFTSV IgG detection was 98% specific, therefore we cannot differentiate between a nonspecific reaction and an asymptomatic infection of SFTSV in 2% of the positive detection. This represent a limitation of the current study, which might also underlie the current minor discrepancy on the significant risk factors that were drawn from IgG and NAb based analysis.

## Conclusions

Based on a cohort of individuals continually sampled with 1-year apart, the anti-SFTSV IgG were determined to be maintained at a stable level, while the NAb level reduced. The high seroprevalence among local residences in endemic region was highlighted, the features of high-risk population as well as their separate risky behavior were explored, which is critical for determining the priority target population for intensive precautions and education measures, as well as optimal allocation of the limited resources available for disease control, such as vaccination if available in the future. It’s supposed that subclinical infection might not provide adequate immunity to protect reinfection of SFTSV for a prolonged period, thus highlighting the ongoing threats of SFTS in endemic regions, which called for an imperative need for vaccine development.

## Supplementary Information

**Additional file 1: Fig. S1. **Location of the study sites, Shangcheng County in Henan Province, and the villages for sampling. The left panel marked the study site of Shangcheng County in Henan Province, the county with the highest SFTS incidence in China. The zoomed map marked the selected villages where the sampling was performed. TM, Tumiao village in Wangqiao town; ZW, Zhaowan village in Wanggang town; PT, Pingtang village in Nianyushan town; HF, Hongfan village Guanmiao town; LTQ, Longtouqiao village in Hefengqiao town. The average annual incidence of each town was marked in red circle and number. **Table S1. **Seroepidemiological questionnaires of SFTS in epidemic area. **Table S2**. Demographic and epidemiological characteristics of participants in the current study. **Table S3**. The risk factors for anti-SFTSV seropositive response for IgG antibody at three sample points by univariate analysis. **Table S4**. The risk factors for anti-SFTSV seropositive response of IgG antibody at three sample points. **Table S5**. The risk factor analysis for anti-SFTSV IgG antibodies seropositive response among the whole population. **Table S6**. The risk factors for anti-SFTSV seropositive response for NAb at three sample points by univariate analysis. **Table S7**. The risk factors for anti-SFTSV seropositive response for NAb by logistic regression model. **Table S8**. The risk factor analysis for anti-SFTSV NAb seropositive response among the adjusted population. **Table S9**. The proportion of the IgG antibody titer in relate to Nab. **Table S10**. The risk factors for anti-SFTSV seropositive response of IgG antibody for different ages people by univariate analysis.

## Data Availability

All data generated and analysed during this study are included in this published article.

## Abbreviations

SFTS: Severe fever with thrombocytopenia
SFTSV: Severe fever with thrombocytopenia Virus
ELISA: Indirect enzyme-linked immunosorbent assay
OD: Optical density
NAb: Neutralization antibody
PRNT50: Plaque reduction neutralization testing
CPE: Cytopathic effect
GMRTs: Geometric mean reciprocal titers
*OR*: Odds ratio
*CI*: Confidence interval
IQR: Interquartile range
