# Supplementary material for: Infection with severe fever with thrombocytopenia virus in healthy population: a cohort study in a high endemic region, China
Source: Infect Dis Poverty. 2021 Nov 16;10:133. doi: 10.1186/s40249-021-00918-0 (PMC8600349; doi:10.1186/s40249-021-00918-0)
Supplement: Supplementary file 1 — Additional file 1: Fig. S1. Location of the study sites, Shangcheng County in Henan Province, and the villages for sampling. The left panel marked the study site of Shangcheng County in Henan Province, the county with the highest SFTS incidence in China. The zoomed map marked the selected villages where the sampling was performed. TM, Tumiao village in Wangqiao town; ZW, Zhaowan village in Wanggang town; PT, Pingtang village in Nianyushan town; HF, Hongfan village Guanmiao town; LTQ, Longtouqiao village in Hefengqiao town. The average annual incidence of each town was marked in red circle and number. Table S1. Seroepidemiological questionnaires of SFTS in epidemic area. Table S2. Demographic and epidemiological characteristics of participants in the current study. Table S3. The risk factors for anti-SFTSV seropositive response for IgG antibody at three sample points by univariate analysis. Table S4. The risk factors for anti-SFTSV seropositive response of IgG antibody at three sample points. Table S5. The risk factor analysis for anti-SFTSV IgG antibodies seropositive response among the whole population. Table S6. The risk factors for anti-SFTSV seropositive response for NAb at three sample points by univariate analysis. Table S7. The risk factors for anti-SFTSV seropositive response for NAb by logistic regression model. Table S8. The risk factor analysis for anti-SFTSV NAb seropositive response among the adjusted population. Table S9. The proportion of the IgG antibody titer in relate to Nab. Table S10. The risk factors for anti-SFTSV seropositive response of IgG antibody for different ages people by univariate analysis. [file 40249_2021_918_MOESM1_ESM.docx]

Appendix to **Infection with Severe fever with thrombocytopenia virus in healthy population: a cohort study in a high endemic region, China**

**Supplemental Table 1. Seroepidemiological questionnaires of SFTS in epidemic area**

Code_________(the same with sample code) Telephone number______________

1. Demographic information
   1. Name_______ Phone number_________________
   2. Gender: (1) Male (2) Female
   3. Birthday_________(mm/yy) (if unclear, fill the actual age________)
   4. Blood pressure__________
   5. Occupation (1) Farming (2) Others_____________
   6. Is there any underlying disease? (1) Hypertension (2) Diabetes (3) Coronary heart disease, CHD (4) Cerebrovascular disease, CVD (5) Anemia (6) Chronic obstructive pulmonary disease, COPD (7) Chronic bronchitis (8) Hepatitis (9) Nephropathy (10) Tumor (11) None (12) Unknown (13) Others_________
   7. Living habit: Smoking? (1) Yes (2) No
   8. Drinking (drinking once per 1-3 days)? (1) Yes (2) No
2. Incidence of SFTS disease

Have you or your family had a tick-borne disease in the last five years?

(1) Myself (2) Family (3) None

If you choose (2), his or her relationship with you_______ Onset date______ Medical institution of clinical visit_____

Have you ever had contact with the confirmed SFTS patient? (1) Yes (2) No (3) Unknown

The way of contact?

(1) Direct contact with patient blood (2) Direct contact with patient secretions and excreta

(3) Treatment / Nursing (4) Stay in the same room (5) Other______________

1. Epidemiological Investigation (Last three months)
   1. Crop planting (1) Rice (2) Corn (3) Peanut (4) Vegetable (5) Rape (6) Sorghum (7) Potato (8) Tea Tree (9) Other_________
   2. Main Farming area (1) Farmland (2) Non-irrigated farmland (3) Tea garden (4) Orchard (5) mountainy forest (6) Other_________
   3. Frequency of agricultural activities in the past 3 months (1) Every day (2) 4-5 times per weeks (3) 2-3 times per weeks (4) ≤1 time per weeks
   4. Do you know ticks? (1) Yes (2) No; If yes, where have you seen it before? _________ Have you ever been bitten by ticks? (1) Yes (2) No (3) Unknown; If yes, bite site______ bite date________; Are there symptoms? (1) Yes (2) No (3) Unknown
   5. Are rats found around the residence? (1) Yes (2) No (3) Unknown
   6. Domestic Animal Feeding in your Home (1) Yes (fill the form below) (2) No (3) Unknown

| Animal type | Number | Contact with animals within 2 weeks | Ticks on animals? |
| --- | --- | --- | --- |
| Dogs |  | (1) Yes (2) No (3) Unknown | (1) Yes (2) No (3) Unknown |
| Cats |  | (1) Yes (2) No (3) Unknown | (1) Yes (2) No (3) Unknown |
| poultries |  | (1) Yes (2) No (3) Unknown | (1) Yes (2) No (3) Unknown |
| Goats |  | (1) Yes (2) No (3) Unknown | (1) Yes (2) No (3) Unknown |
| Cattles |  | (1) Yes (2) No (3) Unknown | (1) Yes (2) No (3) Unknown |
| Pigs |  | (1) Yes (2) No (3) Unknown | (1) Yes (2) No (3) Unknown |

Investigator signature________________ Investigation date_________________

Supplemental Table 2. Demographic and epidemiological characteristics of participants in the current study

| Variables | Pre-epidemic  (*n* = 587) | During-epidemic  (*n* = 350) | Post-epidemic  (*n* = 508) | *P* |
| --- | --- | --- | --- | --- |
| **Age group, years, median (IQR)** | 56 (48–69) | 62 (52–70) | 64 (53–72) | < 0.001 |
| < 50 | 174 (29.6) | 70 (20.0) | 77 (15.2) |  |
| 50–60 | 164 (27.9) | 86 (24.6) | 129 (25.4) |  |
| 60–70 | 115 (19.6) | 94 (26.9) | 132 (26.0) |  |
| ≥ 70 | 134 (22.8) | 100 (28.6) | 170 (33.5) |  |
| **Gender** |  |  |  | 0.561 |
| Male | 156 (26.6) | 101 (28.9) | 149 (29.3) |  |
| Female | 431 (73.4) | 249 (71.1) | 359 (70.7) |  |
| **Underlying diseases*** |  |  |  |  |
| Hypertension |  |  |  |  |
| Yes | 133 (22.7) | 103 (29.4) | 163 (32.1) | 0.002 |
| No | 454 (77.3) | 247 (70.6) | 345 (67.9) |  |
| Diabetes |  |  |  | 0.325 |
| Yes | 13 (2.2) | 10 (2.9) | 19 (3.7) |  |
| No | 574 (97.8) | 340 (97.1) | 489 (96.3) |  |
| Coronary heart disease, CHD | | |  | 0.822 |
| Yes | 43 (7.3) | 28 (8.0) | 43 (8.5) |  |
| No | 544 (92.7) | 322 (92.0) | 465 (91.5) |  |
| Cerebrovascular disease, CVD | |  |  | 0.476 |
| Yes | 10 (1.7) | 7 (2.0) | 14 (2.8) |  |
| No | 577 (98.3) | 343 (98.0) | 494 (97.2) |  |
| Anemia |  |  |  | 0.880 |
| Yes | 18 (3.1) | 10 (2.9) | 13 (2.6) |  |
| No | 569 (96.9) | 340 (97.1) | 495 (97.4) |  |
| Chronic obstructive pulmonary disease, COPD | | | | 0.698 |
| Yes | 9 (1.5) | 8 (2.3) | 10 (2.0) |  |
| No | 578 (98.5) | 342 (97.7) | 498 (98.0) |  |
| Tumor |  |  |  | 0.598 |
| Yes | 17 (2.9) | 8 (2.3) | 10 (2.0) |  |
| No | 570 (97.1) | 342 (97.7) | 498 (98.0) |  |
| **Smoking** |  | | | 0.944 |
| Yes | 86 (14.7) | 51 (14.6) | 79 (15.6) |  |
| No | 501 (85.4) | 299 (85.4) | 429 (84.5) |  |
| **Drinking** |  |  |  | 0.551 |
| Yes | 105 (17.9) | 71 (20.3) | 89 (17.5) |  |
| No | 482 (82.1) | 279 (79.7) | 419 (82.5) |  |
| **Exposure to SFTS patients** | | |  | 0.808 |
| Yes | 14 (2.4) | 10 (2.9) | 11 (2.2) |  |
| No | 573 (97.6) | 340 (97.1) | 497 (97.8) |  |
| **History of tick bite** | | |  | 0.019 |
| Yes | 115 (19.6) | 96 (27.4) | 121 (23.8) |  |
| No | 472 (80.4) | 254 (72.6) | 387 (76.2) |  |
| **Farming frequency in last three months** | | |  | 0.595 |
| ≥ 4/weeks | 286 (48.7) | 177 (50.6) | 239 (47.1) |  |
| < 4/weeks | 301 (51.3) | 173 (49.4) | 269 (53.0) |  |
| **Crops planting in last three months** | | |  |  |
| Vegetables |  |  |  | < 0.001 |
| Yes | 323 (55.0) | 265 (75.7) | 383 (75.4) |  |
| No | 264 (45.0) | 85 (24.3) | 125 (24.6) |  |
| Rapeseed |  |  |  | < 0.001 |
| Yes | 110 (18.7) | 49 (14.0) | 197 (38.8) |  |
| No | 477 (81.3) | 301 (86.0) | 311 (61.2) |  |
| Tea |  |  |  | < 0.001 |
| Yes | 96 (16.4) | 59 (16.9) | 38 (7.5) |  |
| No | 491 (83.7) | 291 (83.1) | 470 (92.5) |  |
| **Forest working** |  |  |  | < 0.001 |
| Yes | 76 (13.0) | 10 (2.9) | 60 (11.8) |  |
| No | 511 (87.1) | 340 (97.1) | 448 (88.2) |  |
| **Close contact with domestic animals in recent two weeks** | | |  |  |
| Dogs |  |  |  | 0.580 |
| Yes | 185 (31.5) | 120 (34.3) | 158 (31.1) |  |
| No | 402 (68.5) | 230 (65.7) | 350 (68.9) |  |
| Cats |  |  |  | 0.017 |
| Yes | 47 (8.0) | 31 (8.9) | 66 (13.0) |  |
| No | 540 (92.0) | 319 (91.1) | 442 (87.0) |  |
| Poultry |  |  |  | 0.066 |
| Yes | 171 (29.1) | 120 (34.3) | 137 (27.0) |  |
| No | 416 (70.9) | 230 (65.7) | 371 (73.0) |  |
| Goats |  |  |  | 0.234 |
| Yes | 5 (0.9) | 0 (0.00) | 3 (0.6) |  |
| No | 582 (99.2) | 350 (100.0) | 505 (99.4) |  |
| Cattle |  |  |  | 0.856 |
| Yes | 6 (1.0) | 5 (1.4) | 6 (1.2) |  |
| No | 581 (99.0) | 345 (98.6) | 502 (98.8) |  |
| Pigs |  |  |  | 0.033 |
| Yes | 40 (6.8) | 37 (10.6) | 56 (11.0) |  |
| No | 547 (93.2) | 313 (89.4) | 452 (89.0) |  |
| **Seropositive rate** |  |  |  |  |
| IgG antibody | 70 (11.9) | 47 (13.4) | 80 (15.8) | 0.183 |
| Neutralizing antibody | 40 (6.8) | 27 (7.7) | 50 (9.8) | 0.178 |

Note: The data were presented as *n* (rate, %) until otherwise denoted. IQR: interquartile range. The P value was calculated by performing chi-square test or Fisher exact test. Because of the small number of participants who had the underlying diseases, including chronic bronchitis, hepatitis, nephropathy, these variables were not analyzed.

Supplemental Table 3. The risk factors for anti-SFTSV seropositive response for IgG antibody at three sample points by univariate analysis.

| Variables | Pre-epidemic (*n* = 587) | | |  | During-epidemic (*n* = 350) | | |  | Post-epidemic (*n* = 508) | | |  |
| --- | --- | --- | --- | --- | --- | --- | --- | --- | --- | --- | --- | --- |
|  | Positive (rate, %) | *OR* (95%*CI*) | *P* |  | Positive (rate, %) | *OR* (95%*CI*) | *P* |  | Positive (rate, %) | *OR* (95%*CI*) | *P* |  |
| **Age group, years** | | | |  |  |  |  |  |  |  |  |  |
| < 50 | 13 (7.5) | Reference |  |  | 5 (7.1) | Reference |  |  | 6 (7.8) | Reference |  |  |
| 50–60 | 12 (7.3) | 0.978 (0.433, 2.210) | 0.957 |  | 11 (12.8) | 1.907 (0.630, 5.774) | 0.254 |  | 20 (15.5) | 2.171 (0.831, 5.671) | 0.113 |  |
| 60–70 | 19 (16.5) | 2.451 (1.159, 5.186) | 0.019 |  | 11 (11.7) | 1.723 (0.570, 5.206) | 0.335 |  | 19 (14.4) | 1.990 (0.758, 5.221) | 0.162 |  |
| ≥ 70 | 26 (19.4) | 2.981 (1.467, 6.058) | 0.003 |  | 20 (20.0) | 3.250 (1.157, 9.133) | 0.025 |  | 35 (20.6) | 3.068 (1.232, 7.640) | 0.016 |  |
| **Gender** |  |  |  |  |  |  |  |  |  |  |  |  |
| Female | 54 (12.5) | 1.253 (0.694, 2.262) | 0.454 |  | 35 (14.1) | 1.213 (0.602, 2.444) | 0.589 |  | 60 (16.7) | 1.294 (0.749, 2.236) | 0.355 |  |
| Male | 16 (10.3) | Reference |  |  | 12 (11.9) | Reference |  |  | 20 (13.4) | Reference |  |  |
| **Underlying diseases** | | | |  |  |  |  |  |  |  |  |  |
| Hypertension | |  |  |  |  |  |  |  |  |  |  |  |
| Yes | 21 (15.8) | 1.55 (0.892, 2.692) | 0.120 |  | 15 (14.6) | 1.145 (0.591, 2.219) | 0.688 |  | 29 (17.8) | 1.248 (0.757, 2.056) | 0.385 |  |
| No | 49 (10.8) | Reference |  |  | 32 (13.0) | Reference |  |  | 51 (14.8) | Reference |  |  |
| Diabetes |  |  |  |  |  |  |  |  |  |  |  |  |
| Yes | 3 (23.1) | 2.27 (0.609, 8.457) | 0.222 |  | 3 (30.0) | 2.883 (0.719, 11.565) | 0.135 |  | 3 (15.8) | 1.003 (0.285, 3.526) | 0.996 |  |
| No | 67 (11.7) | Reference |  |  | 44 (12.9) | Reference |  |  | 77 (15.8) | Reference |  |  |
| Coronary heart disease, CHD | | |  |  |  |  |  |  |  |  |  |  |
| Yes | 5 (14.5) | 1.249 (0.468, 3.332) | 0.657 |  | 3 (12.5) | 0.916 (0.262, 3.198) | 0.89 |  | 3 (10.0) | 0.579 (0.171, 1.955) | 0.378 |  |
| No | 65 (11.8) | Reference |  |  | 44 (13.5) | Reference |  |  | 77 (16.1) | Reference |  |  |
| Cerebrovascular disease, CVD | | | | | |  |  |  |  |  |  |  |
| Yes | 2 (20.0) | 1.871 (0.389, 8.995) | 0.434 |  | 1 (14.3) | 1.076 (0.127, 9.143) | 0.946 |  | 2 (14.3) | 0.889 (0.195, 4.049) | 0.879 |  |
| No | 68 (11.8) | Reference |  |  | 46 (13.4) | Reference |  |  | 78 (15.8) | Reference |  |  |
| Anemia |  |  |  |  |  |  |  |  |  |  |  |  |
| Yes | 3 (16.7) | 1.499 (0.423, 5.312) | 0.531 |  | 0 (0.0) | - | - |  | 1 (7.7) | 0.439 (0.056, 3.423) | 0.432 |  |
| No | 67 (11.8) | Reference |  |  | 47 (13.8) |  |  |  | 79 (16.0) | Reference |  |  |
| Chronic obstructive pulmonary disease, COPD | | | | | |  |  |  |  |  |  |  |
| Yes | 4 (44.4) | 6.206 (1.626, 23.69) | 0.008 |  | 2 (25.0) | 2.2 (0.431, 11.237) | 0.343 |  | 2 (20.0) | 1.346 (0.281, 6.459) | 0.710 |  |
| No | 66 (11.4) | Reference |  |  | 45 (13.2) | Reference |  |  | 78 (15.7) | Reference |  |  |
| Tumor |  |  |  |  |  |  |  |  |  |  |  |  |
| Yes | 0 (0.00) | - | - |  | 0 (0.00) | - | - |  | 2 (20.00) | 1.346 (0.281, 6.459) | 0.710 |  |
| No | 70 (12.28) |  |  |  |  |  |  |  | 78 (15.66) | Reference |  |  |
| **Smoking** |  |  |  |  |  |  |  |  |  |  |  |  |
| Yes | 8 (9.3) | 0.726 (0.335, 1.576) | 0.418 |  | 8 (15.7) | 1.24 (0.543, 2.834) | 0.609 |  | 11 (13.9) | 0.844 (0.425, 1.678) | 0.628 |  |
| No | 62 (12.4) | Reference |  |  | 39 (13.0) | Reference |  |  | 69 (16.1) | Reference |  |  |
| **Drinking** |  |  |  |  |  |  |  |  |  |  |  |  |
| Yes | 10 (9.5) | 0.74 (0.366, 1.499) | 0.404 |  | 12 (16.9) | 1.418 (0.694, 2.897) | 0.338 |  | 14 (15.7) | 0.998 (0.533, 1.871) | 0.996 |  |
| No | 60 (12.5) | Reference |  |  | 35 (12.5) | Reference |  |  | 66 (15.8) | Reference |  |  |
| **Exposure to SFTS patients** | | |  |  |  |  |  |  |  |  |  |  |
| Yes | 0 (0.0) | - | - |  | 2 (20.0) | 1.639 (0.337, 7.964) | 0.540 |  | 1 (9.1) | 0.529 (0.067, 4.192) | 0.547 |  |
| No | 70 (12.2) |  |  |  | 45 (13.2) | Reference |  |  | 79 (15.9) | Reference |  |  |
| **History of tick bite** | |  | | | |  |  |  |  |  |  |  |
| Yes | 21 (18.3) | 1.929 (1.104, 3.369) | 0.021 |  | 20 (20.8) | 2.212 (1.174, 4.170) | 0.014 |  | 23 (19.0) | 1.359 (0.797, 2.318) | 0.261 |  |
| No | 49 (10.4) | Reference |  |  | 27 (10.6) | Reference |  |  | 57 (14.7) | Reference |  |  |
| **Farming frequency in last three months** | | | | | |  |  |  |  |  |  |  |
| ≥4/weeks | 42 (14.7) | 1.678 (1.009, 2.790) | 0.046 |  | 32 (18.1) | 2.325 (1.209, 4.468) | 0.011 |  | 40 (16.7) | 1.151 (0.714, 1.855) | 0.564 |  |
| <4/weeks | 28 (9.3) | Reference |  |  | 15 (8.7) | Reference |  |  | 40 (14.9) | Reference |  |  |
| **Crops planting in last three months** | | | |  |  |  |  |  |  |  |  |  |
| Vegetables |  |  |  |  |  |  |  |  |  |  |  |  |
| Yes | 45 (13.9) | 1.547 (0.921, 2.599) | 0.099 |  | 41 (15.5) | 2.410 (0.985, 5.894) | 0.054 |  | 65 (17.0) | 1.499 (0.821, 2.736) | 0.187 |  |
| No | 25 (9.5) | Reference |  |  | 6 (7.1) | Reference |  |  | 15 (12.0) | Reference |  |  |
| Rapeseed |  |  |  |  |  |  |  |  |  |  |  |  |
| Yes | 18 (16.4) | 1.599 (0.894, 2.860) | 0.114 |  | 10 (20.4) | 1.83 (0.843, 3.972) | 0.127 |  | 37 (18.8) | 1.441 (0.891, 2.332) | 0.136 |  |
| No | 52 (10.9) | Reference |  |  | 37 (12.3) | Reference |  |  | 43 (13.8) | Reference |  |  |
| Tea |  |  |  |  |  |  |  |  |  |  |  |  |
| Yes | 16 (16.0) | 1.527 (0.834, 2.796) | 0.170 |  | 20 (22.0) | 2.420 (1.281, 4.574) | 0.006 |  | 7 (14.9) | 0.930 (0.401, 2.157) | 0.866 |  |
| No | 54 (11.1) | Reference |  |  | 27 (10.4) | Reference |  |  | 73 (15.8) | Reference |  |  |
| **Forest working** | |  | | | |  |  |  |  |  |  |  |
| Yes | 9 (11.8) | 0.991 (0.470, 2.088) | 0.981 |  | 3 (30.0) | 2.883 (0.719, 11.565) | 0.135 |  | 10 (16.7) | 1.080 (0.523, 2.231) | 0.835 |  |
| No | 61 (11.9) | Reference |  |  | 44 (12.9) | Reference |  |  | 70 (15.6) | Reference |  |  |
| **Close contact with domestic animals in recent two weeks** | | | | | |  |  |  |  |  |  |  |
| Dogs |  |  |  |  |  |  |  |  |  |  |  |  |
| Yes | 23 (12.4) | 1.072 (0.630, 1.826) | 0.797 |  | 23 (19.2) | 2.035 (1.094, 3.786) | 0.025 |  | 27 (17.1) | 1.155 (0.696, 1.918) | 0.578 |  |
| No | 47 (11.7) | Reference |  |  | 24 (10.4) | Reference |  |  | 53 (15.1) | Reference |  |  |
| Cats |  |  |  |  |  |  |  |  |  |  |  |  |
| Yes | 8 (17.0) | 1.581 (0.707, 3.539) | 0.265 |  | 11 (35.5) | 4.324 (1.917, 9.752) | <0.001 |  | 16 (24.2) | 1.890 (1.015, 3.521) | 0.045 |  |
| No | 62 (11.5) | Reference |  |  | 36 (11.3) | Reference |  |  | 64 (14.5) | Reference |  |  |
| Poultry |  |  |  |  |  |  |  |  |  |  |  |  |
| Yes | 23 (13.5) | 1.220 (0.715, 2.081) | 0.465 |  | 21 (17.5) | 1.664 (0.893, 3.104) | 0.109 |  | 24 (17.5) | 1.195 (0.707, 2.018) | 0.506 |  |
| No | 47 (11.3) | Reference |  |  | 26 (11.3) | Reference |  |  | 56 (15.1) | Reference |  |  |
| Goats |  |  |  |  |  |  |  |  |  |  |  |  |
| Yes | 2 (40.0) | 5.039 (0.827, 30.699) | 0.079 |  | 0 (0.0) | - | - |  | 2 (66.7) | 10.949 (0.981, 122.214) | 0.052 |  |
| No | 68 (11.7) | Reference |  |  | 47 (13.4) |  |  |  | 78 (15.5) | Reference |  |  |
| Cattle |  |  |  |  |  |  |  |  |  |  |  |  |
| Yes | 1 (16.7) | 1.484 (0.171, 12.890) | 0.720 |  | 1 (20.0) | 1.625 (0.178, 14.86) | 0.667 |  | 2 (33.3) | 2.718 (0.489, 15.095) | 0.253 |  |
| No | 69 (11.9) | Reference |  |  | 46 (13.3) | Reference |  |  | 78 (15.5) | Reference |  |  |
| Pigs |  |  |  |  |  |  |  |  |  |  |  |  |
| Yes | 6 (15.0) | 1.332 (0.538, 3.296) | 0.535 |  | 6 (16.2) | 1.284 (0.505, 3.267) | 0.600 |  | 11 (19.6) | 1.357 (0.669, 2.752) | 0.398 |  |
| No | 64 (11.7) | Reference |  |  | 41 (13.1) | Reference |  |  | 69 (15.3) | Reference |  |  |

Note: OR: odds ratio; CI: confidence interval;.

Supplemental Table 4. The risk factors for anti-SFTSV seropositive response of IgG antibody at three sample points

| Variables | Pre-epidemic | |  | During-epidemic | |  | Post-epidemic | |
| --- | --- | --- | --- | --- | --- | --- | --- | --- |
|  | *OR* (95%*CI*) | *P* |  | *OR* (95%*CI*) | *P* |  | *OR* (95%*CI*) | *P* |
| **Age group, years** |  |  |  |  |  |  |  |  |
| < 50 | Reference |  |  | Reference |  |  | Reference |  |
| 50–60 | 0.897 (0.390–2.062) | 0.798 |  | 1.822 (0.0568–5.841) | 0.313 |  | 2.200 (0.834–5.804) | 0.111 |
| 60–70 | 2.013 (0.932–4.351) | 0.075 |  | 1.459 (0.457–4.657) | 0.523 |  | 1.587 (0.586–4.294) | 0.364 |
| ≥ 70 | 2.430 (1.171–5.043) | 0.017 |  | 3.227 (1.086–9.588) | 0.035 |  | 2.885 (1.148–7.254) | 0.024 |
| **Close contact with cats in recent two weeks** |  |  |  | 2.858 (1.179–6.930) | 0.020 |  |  |  |

Supplemental Table 5. The risk factor analysis for anti-SFTSV IgG antibodies seropositive response among the whole population

| Variables | Total number  (Composition ratio, %) | Number of positive  (rate, %) | Univariate analysis | |  | Multivariate analysis | |
| --- | --- | --- | --- | --- | --- | --- | --- |
|  |  |  | *OR* (95% *CI*) | *P* |  | *OR* (95% *CI*) | *P* |
| **Age group, years, median (IQR)** | 58 (49–70) |  | 1.330 (1.114–1.587) | 0.002 |  | 1.315 (1.092–1.584) | 0.004 |
| < 50 | 222 (25.1) | 18 (8.1) | Reference |  |  | Reference |  |
| 50–60 | 236 (26.6) | 28 (11.9) | 1.526 (0.818–2.844) | 0.184 |  | 1.510 (0.799–2.854) | 0.205 |
| 60–70 | 189 (21.3) | 26 (13.8) | 1.808 (0.958–3.412) | 0.068 |  | 1.599 (0.832–3.075) | 0.159 |
| ≥ 70 | 239 (27.0) | 43 (18.0) | 2.486 (1.386–4.459) | 0.002 |  | 2.440 (1.334–4.461) | 0.004 |
| **Gender** |  |  |  |  |  |  |  |
| Female | 646 (72.9) | 89 (13.8) | 1.315 (0.827–2.093) | 0.248 |  |  |  |
| Male | 240 (27.1) | 26 (10.8) | Reference |  |  |  |  |
| **Underlying diseases** |  |  |  |  |  |  |  |
| Hypertension |  |  |  |  |  |  |  |
| Yes | 218 (24.6) | 35 (16.1) | 1.406 (0.914–2.162) | 0.121 |  |  |  |
| No | 668 (75.4) | 80 (12.0) | Reference |  |  |  |  |
| Diabetes |  |  |  |  |  |  |  |
| Yes | 27 (3.0) | 5 (18.5) | 1.548 (0.574–4.171) | 0.388 |  |  |  |
| No | 859 (97.0) | 110 (12.8) | Reference |  |  |  |  |
| Coronary heart disease, CHD | |  |  |  |  |  |  |
| Yes | 53 (6.0) | 6 (11.3) | 0.848 (0.354–2.031) | 0.711 |  |  |  |
| No | 833 (94.0) | 109 (13.1) | Reference |  |  |  |  |
| Cerebrovascular disease, CVD | |  |  |  |  |  |  |
| Yes | 17 (1.9) | 2 (11.8) | 0.892 (0.201–3.952) | 0.880 |  |  |  |
| No | 869 (98.1) | 113 (13.0) | Reference |  |  |  |  |
| Anemia |  |  |  |  |  |  |  |
| Yes | 24 (2.7) | 1 (4.2) | 0.285 (0.038–2.133) | 0.222 |  |  |  |
| No | 862 (97.3) | 114 (13.2) | Reference |  |  |  |  |
| Chronic obstructive pulmonary disease, COPD | | |  |  |  |  |  |
| Yes | 16 (1.8) | 4 (25.0) | 2.279 (0.722–7.191) | 0.160 |  |  |  |
| No | 870 (98.2) | 111 (12.8) | Reference |  |  |  |  |
| Tumor |  |  |  |  |  |  |  |
| Yes | 21 (2.4) | 2 (9.5) | 0.701 (0.161–3.048) | 0.635 |  |  |  |
| No | 865 (97.6) | 113 (13.1) | Reference |  |  |  |  |
| **Smoking** |  |  |  |  |  |  |  |
| Yes | 126 (14.2) | 13 (10.3) | 0.742 (0.403–1.367) | 0.339 |  |  |  |
| No | 760 (85.8) | 102 (13.4) | Reference |  |  |  |  |
| **Drinking** |  |  |  |  |  |  |  |
| Yes | 147 (16.6) | 20 (13.6) | 1.068 (0.636–1.793) | 0.805 |  |  |  |
| No | 739 (83.4) | 95 (12.9) | Reference |  |  |  |  |
| **Exposure to SFTS patients** | |  |  |  |  |  |  |
| Yes | 22 (2.5) | 2 (9.1) | 0.665 (0.153–2.882) | 0.585 |  |  |  |
| No | 864 (97.5) | 113 (13.1) | Reference |  |  |  |  |
| **History of tick bite** | |  |  |  |  |  |  |
| Yes | 176 (19.9) | 32 (18.2) | 1.679 (1.074–2.623) | 0.023 |  | 1.399 (0.87–2.25) | 0.166 |
| No | 710 (80.1) | 83 (11.7) | Reference |  |  | Reference |  |
| **Farming frequency in last three months** | | |  |  |  |  |  |
| ≥ 4/weeks | 396 (44.7) | 65 (16.4) | 1.728 (1.164–2.566) | 0.007 |  | 1.356 (0.853–2.154) | 0.197 |
| < 4/weeks | 490 (55.3) | 50 (10.2) | Reference |  |  | Reference |  |
| **Crops planting in last three months** | | |  |  |  |  |  |
| Vegetables |  |  |  |  |  |  |  |
| Yes | 650 (73.4) | 90 (13.9) | 1.356 (0.847–2.171) | 0.204 |  |  |  |
| No | 236 (26.6) | 25 (10.6) | Reference |  |  |  |  |
| Rapeseed |  |  |  |  |  |  |  |
| Yes | 394 (44.5) | 64 (16.2) | 1.677 (1.130–2.488) | 0.010 |  | 1.197 (0.750–1.910) | 0.451 |
| No | 492 (55.5) | 51 (10.4) | Reference |  |  | Reference |  |
| Tea |  |  |  |  |  |  |  |
| Yes | 136 (15.4) | 29 (21.3) | 2.093 (1.311–3.341) | 0.002 |  | 1.698 (1.002–2.880) | 0.049 |
| No | 750 (84.6) | 86 (11.5) | Reference |  |  | Reference |  |
| **Forest working** |  |  |  |  |  |  |  |
| Yes | 60 (6.8) | 8 (13.3) | 1.034 (0.478–2.236) | 0.933 |  |  |  |
| No | 826 (93.2) | 107 (13.0) | Reference |  |  |  |  |
| **Close contact with domestic animals in recent two weeks** | |  |  |  |  |  |  |
| Dogs |  |  |  |  |  |  |  |
| Yes | 288 (32.5) | 38 (13.2) | 1.028 (0.678–1.560) | 0.895 |  |  |  |
| No | 598 (67.5) | 77 (12.9) | Reference |  |  |  |  |
| Cats |  |  |  |  |  |  |  |
| Yes | 86 (9.7) | 23 (26.7) | 2.81 (1.663–4.747) | <0.001 |  | 2.195 (1.261–3.818) | 0.005 |
| No | 800 (90.3) | 92 (11.5) | Reference |  |  | Reference |  |
| Poultry |  |  |  |  |  |  |  |
| Yes | 254 (28.7) | 41 (16.1) | 1.451 (0.96–2.194) | 0.077 |  | 1.156 (0.742–1.799) | 0.522 |
| No | 632 (71.3) | 74 (11.7) | Reference |  |  | Reference |  |
| Goats |  |  |  |  |  |  |  |
| Yes | 4 (0.5) | 2 (50.0) | 6.805 (0.949–48.793) | 0.056 |  | 5.127 (0.678–38.755) | 0.113 |
| No | 882 (99.5) | 113 (12.8) | Reference |  |  | Reference |  |
| Cattle |  |  |  |  |  |  |  |
| Yes | 11 (1.2) | 3 (27.3) | 2.555 (0.668–9.772) | 0.171 |  |  |  |
| No | 875 (98.8) | 112 (12.8) | Reference |  |  |  |  |
| Pigs |  |  |  |  |  |  |  |
| Yes | 72 (8.1) | 13 (18.1) | 1.538 (0.815–2.903) | 0.184 |  |  |  |
| No | 814 (91.9) | 102 (12.5) | Reference |  |  |  |  |

Supplemental Table 6. The risk factors for anti-SFTSV seropositive response for NAb at three sample points by univariate analysis.

| Variables | Pre-epidemic (*n* = 587) | | |  | During-epidemic in 2018 (*n* = 350) | | |  | Post-epidemic in 2018 (*n* = 508) | | |
| --- | --- | --- | --- | --- | --- | --- | --- | --- | --- | --- | --- |
|  | Positive (rate, %) | *OR* (95%*CI*) | *P* |  | Positive (rate, %) | *OR* (95%*CI*) | *P* |  | Positive (rate, %) | *OR* (95%*CI*) | *P* |
| **Age group, years** | | | | | |  |  |  |  |  |  |
| < 50 | 8 (4.6) | Reference |  |  | 2 (2.9) | Reference |  |  | 4 (5.2) | Reference |  |
| 50–60 | 7 (4.3) | 0.925 (0.328, 2.611) | 0.883 |  | 5 (5.8) | 2.099 (0.395, 11.162) | 0.385 |  | 12 (9.3) | 1.872 (0.582, 6.023) | 0.293 |
| 60–70 | 12 (10.4) | 2.417 (0.956, 6.113) | 0.062 |  | 7 (7.5) | 2.736 (0.551, 13.593) | 0.219 |  | 11 (8.3) | 1.659 (0.509, 5.403) | 0.401 |
| ≥ 70 | 13 (9.7) | 2.229 (0.896, 5.546) | 0.085 |  | 13 (13.0) | 5.080 (1.109, 23.278) | 0.036 |  | 23 (13.5) | 2.855 (0.952, 8.563) | 0.061 |
| **Gender** | | | |  |  |  |  |  |  |  |  |
| Female | 31 (7.2) | 1.266 (0.589, 2.723) | 0.546 |  | 20 (8.0) | 1.173 (0.480, 2.866) | 0.727 |  | 41 (11.4) | 2.006 (0.949, 4.239) | 0.068 |
| Male | 9 (5.8) | Reference |  |  | 7 (6.9) | Reference |  |  | 9 (6.0) | Reference |  |
| **Underlying diseases** | | | | | |  |  |  |  |  |  |
| Hypertension | | | |  |  |  |  |  |  |  |  |
| Yes | 13 (9.8) | 1.713 (0.858, 3.422) | 0.127 |  | 8 (7.8) | 1.011 (0.428, 2.388) | 0.981 |  | 20 (12.3) | 1.469 (0.806, 2.674) | 0.209 |
| No | 27 (6.0) | Reference |  |  | 19 (7.7) | Reference |  |  | 30 (8.7) | Reference |  |
| Diabetes |  |  |  |  |  |  |  |  |  |  |  |
| Yes | 1 (7.7) | 1.143 (0.145, 9.021) | 0.899 |  | 2 (20.0) | 3.15 (0.635, 15.635) | 0.160 |  | 3 (15.8) | 1.763 (0.496, 6.274) | 0.381 |
| No | 39 (6.8) | Reference |  |  | 25 (7.4) | Reference |  |  | 47 (9.6) | Reference |  |
| Coronary heart disease, CHD | | |  |  |  |  |  |  |  |  |  |
| Yes | 3 (8.6) | 1.305 (0.382, 4.462) | 0.671 |  | 1 (4.2) | 0.502 (0.065, 3.865) | 0.508 |  | 2 (6.7) | 0.640 (0.148, 2.77) | 0.550 |
| No | 37 (6.7) | Reference |  |  | 26 (8.0) | Reference |  |  | 48 (10.0) | Reference |  |
| Cerebrovascular disease, CVD | | |  |  |  |  |  |  |  |  |  |
| Yes | 1 (10.0) | 1.533 (0.189, 12.41) | 0.689 |  | 1 (14.3) | 2.032 (0.236, 17.522) | 0.519 |  | 2 (14.3) | 1.549 (0.337, 7.125) | 0.574 |
| No | 39 (6.8) | Reference |  |  | 26 (7.6) | Reference |  |  | 48 (9.7) | Reference |  |
| Anemia |  |  |  |  |  |  |  |  |  |  |  |
| Yes | 2 (11.1) | 1.747 (0.387, 7.878) | 0.468 |  | 0 (0.0) | - | - |  | 2 (15.4) | 1.693 (0.365, 7.865) | 0.502 |
| No | 38 (6.7) | Reference |  |  | 27 (7.9) |  |  |  | 48 (9.7) | Reference |  |
| Chronic obstructive pulmonary disease, COPD | | |  |  |  |  |  |  |  |  |  |
| Yes | 2 (22.2) | 4.060 (0.815, 20.221) | 0.087 |  | 2 (25.0) | 4.227 (0.811, 22.037) | 0.087 |  | 0 (0.0) | - | - |
| No | 38 (6.6) | Reference |  |  | 25 (7.3) | Reference |  |  | 50 (10.0) |  |  |
| Tumor |  |  |  |  |  |  |  |  |  |  |  |
| Yes | 0 (0.0) | - | - |  | 0 (0.0) | - | - |  | 1 (10.0) | 1.018 (0.126, 8.206) | 0.987 |
| No | 40 (7.0) |  |  |  | 27 (7.9) |  |  |  | 49 (9.8) | Reference |  |
| **Smoking** |  |  |  |  |  |  |  |  |  |  |  |
| Yes | 4 (4.7) | 0.630 (0.218, 1.817) | 0.393 |  | 5 (9.8) | 1.369 (0.494, 3.795) | 0.547 |  | 6 (7.6) | 0.719 (0.296, 1.749) | 0.467 |
| No | 36 (7.2) | Reference |  |  | 22 (7.4) | Reference |  |  | 44 (10.3) | Reference |  |
| **Drinking** |  |  |  |  |  |  |  |  |  |  |  |
| Yes | 6 (5.7) | 0.799 (0.326, 1.954) | 0.622 |  | 9 (12.7) | 2.105 (0.903, 4.908) | 0.085 |  | 7 (7.9) | 0.746 (0.324, 1.718) | 0.492 |
| No | 34 (7.1) | Reference |  |  | 18 (6.5) | Reference |  |  | 43 (10.3) | Reference |  |
| **Exposure to SFTS patients** | | |  |  |  |  |  |  |  |  |  |
| Yes | 0 (0.0) | - | - |  | 1 (10.0) | 1.342 (0.164, 11.006) | 0.784 |  | 1 (9.1) | 0.914 (0.115, 7.294) | 0.933 |
| No | 40 (7.0) | Reference |  |  | 26 (7.7) | Reference |  |  | 49 (9.9) | Reference |  |
| **History of tick bite** | | | |  |  |  |  |  |  |  |  |
| Yes | 9 (7.8) | 1.208 (0.558, 2.613) | 0.632 |  | 8 (8.3) | 1.124 (0.475, 2.661) | 0.790 |  | 17 (14.1) | 1.753 (0.939, 3.275) | 0.078 |
| No | 31 (6.6) | Reference |  |  | 19 (7.5) | Reference |  |  | 33 (8.5) | Reference |  |
| **Farming frequency in last three months** | | | |  |  |  |  |  |  |  |  |
| ≥4/weeks | 23 (8.0) | 1.461 (0.764, 2.796) | 0.252 |  | 17 (9.6) | 1.732 (0.770, 3.897) | 0.184 |  | 24 (10.0) | 1.043 (0.582, 1.872) | 0.887 |
| <4/weeks | 17 (5.7) | Reference |  |  | 10 (5.8) | Reference |  |  | 26 (9.7) | Reference |  |
| **Crops planting in last three months** | | | | | |  |  |  |  |  |  |
| Vegetables |  |  |  |  |  |  |  |  |  |  |  |
| Yes | 27 (8.4) | 1.761 (0.890, 3.486) | 0.104 |  | 25 (9.4) | 4.323 (1.002, 18.647) | 0.050 |  | 39 (10.2) | 1.175 (0.582, 2.37) | 0.653 |
| No | 13 (4.9) | Reference |  |  | 2 (2.4) | Reference |  |  | 11 (8.8) | Reference |  |
| Rapeseed |  |  |  |  |  |  |  |  |  |  |  |
| Yes | 12 (10.9) | 1.964 (0.965, 3.997) | 0.063 |  | 5 (10.2) | 1.441 (0.519, 4.004) | 0.483 |  | 22 (11.2) | 1.271 (0.705, 2.291) | 0.426 |
| No | 28 (5.9) | Reference |  |  | 22 (7.3) | Reference |  |  | 28 (9.0) | Reference |  |
| Tea |  |  |  |  |  |  |  |  |  |  |  |
| Yes | 8 (8.0) | 1.236 (0.552, 2.770) | 0.606 |  | 7 (7.7) | 0.996 (0.407, 2.439) | 0.993 |  | 5 (10.6) | 1.101 (0.414, 2.923) | 0.848 |
| No | 32 (6.6) | Reference |  |  | 20 (7.7) | Reference |  |  | 45 (9.8) | Reference |  |
| **Forest working** | | | |  |  |  |  |  |  |  |  |
| Yes | 7 (9.2) | 1.469 (0.626, 3.451) | 0.377 |  | 1 (10.0) | 1.342 (0.164, 11.006) | 0.784 |  | 5 (8.3) | 0.814 (0.310, 2.139) | 0.677 |
| No | 33 (6.5) | Reference |  |  | 26 (7.7) | Reference |  |  | 45 (10.0) | Reference |  |
| **Close contact with domestic animals in recent two weeks** | | | | | |  |  |  |  |  |  |
| Dogs |  |  |  |  |  |  |  |  |  |  |  |
| Yes | 13 (7.0) | 1.050 (0.529, 2.084) | 0.890 |  | 12 (10.0) | 1.593 (0.720, 3.521) | 0.250 |  | 16 (10.1) | 1.047 (0.560, 1.959) | 0.885 |
| No | 27 (6.7) | Reference |  |  | 15 (6.5) | Reference |  |  | 34 (9.7) | Reference |  |
| Cats |  |  |  |  |  |  |  |  |  |  |  |
| Yes | 6 (12.8) | 2.178 (0.864, 5.489) | 0.099 |  | 9 (29.0) | 6.841 (2.754, 16.991) | <0.001 |  | 10 (15.2) | 1.795 (0.850, 3.789) | 0.125 |
| No | 34 (6.3) | Reference |  |  | 18 (5.6) | Reference |  |  | 40 (9.1) | Reference |  |
| Poultry |  |  |  |  |  |  |  |  |  |  |  |
| Yes | 10 (5.9) | 0.799 (0.382, 1.673) | 0.552 |  | 11 (9.2) | 1.350 (0.606, 3.009) | 0.463 |  | 12 (8.8) | 0.841 (0.426, 1.662) | 0.619 |
| No | 30 (7.2) | Reference |  |  | 16 (7.0) |  |  |  | 38 (10.2) | Reference |  |
| Goats |  |  |  |  |  |  |  |  |  |  |  |
| Yes | 1 (20.0) | 3.481 (0.380, 31.897) | 0.270 |  | 0 (0.0) | - | - |  | 1 (33.3) | 4.653 (0.414, 52.248) | 0.213 |
| No | 39 (6.7) | Reference |  |  | 27 (7.7) |  |  |  | 49 (9.7) | Reference |  |
| cattle |  |  |  |  |  |  |  |  |  |  |  |
| Yes | 0 (0.0) | - | - |  | 1 (20.0) | 3.067 (0.331, 28.453) | 0.324 |  | 2 (33.3) | 4.729 (0.844, 26.496) | 0.077 |
| No | 40 (6.9) |  |  |  | 26 (7.5) | Reference |  |  | 48 (9.6) | Reference |  |
| Pigs |  |  |  |  |  |  |  |  |  |  |  |
| Yes | 4 (10.0) | 1.577 (0.532, 4.676) | 0.411 |  | 3 (8.1) | 1.063 (0.304, 3.715) | 0.924 |  | 6 (10.7) | 1.113 (0.451, 2.743) | 0.816 |
| No | 36 (6.6) | Reference |  |  | 24 (7.7) | Reference |  |  | 44 (9.7) | Reference |  |

Supplemental Table 7. The risk factors for anti-SFTSV seropositive response for NAb by logistic regression model.

| Variables | During-epidemic | |  | Post-epidemic | |
| --- | --- | --- | --- | --- | --- |
|  | *OR* (95% *CI*) | *P* |  | *OR* (95% *CI*) | *P* |
| **Age, years** |  |  |  |  |  |
| < 50 |  |  |  | Reference |  |
| 50–60 |  |  |  | 2.138 (0.808–5.661) | 0.130 |
| 60–70 |  |  |  | 1.499 (0.551–4.077) | 0.430 |
| ≥ 70 |  |  |  | 2.914 (1.152–7.370) | 0.020 |
| **Close contact with cats in recent two weeks** | 5.987 (2.318–15.464) | < 0.001 |  |  |  |

Supplemental Table 8. The risk factor analysis for anti-SFTSV NAb seropositive response among the adjusted population

| Variables | Total number | Number of positive | Univariate analysis | |  | Multivariate analysis | |
| --- | --- | --- | --- | --- | --- | --- | --- |
|  | (Composition ratio, %) | (rate, %) | *OR* (95% *CI*) | *P* |  | *OR* (95% *CI*) | *P* |
| **Age group, years, median (IQR)** | 59 (50–71) |  | 1.486 (1.184–1.864) | 0.001 |  | 1.413 (1.123-1.777) | 0.003 |
| < 50 | 222 (25.1) | 10 (4.5) | Reference |  |  | Reference |  |
| 50–60 | 236 (26.6) | 13 (5.5) | 1.236 (0.531–2.879) | 0.624 |  | 1.259 (0.539–2.939) | 0.594 |
| 60–70 | 189 (21.3) | 17 (9.0) | 2.095 (0.935–4.694) | 0.072 |  | 1.989 (0.884–4.475) | 0.096 |
| ≥ 70 | 239 (27.0) | 30 (12.6) | 3.043 (1.451–6.383) | 0.003 |  | 2.691 (1.271-5.695) | 0.010 |
| **Gender** |  |  |  |  |  |  |  |
| Female | 646 (72.9) | 56 (8.7) | 1.532 (0.836–2.807) | 0.167 |  |  |  |
| Male | 240 (27.1) | 14 (5.8) | Reference |  |  |  |  |
| **Underlying diseases** |  |  |  |  |  |  |  |
| Hypertension |  |  |  |  |  |  |  |
| Yes | 218 (24.6) | 22 (10.1) | 1.45 (0.854–2.462) | 0.169 |  |  |  |
| No | 668 (75.4) | 48 (7.2) | Reference |  |  |  |  |
| Diabetes |  |  |  |  |  |  |  |
| Yes | 27 (3.0) | 4 (14.8) | 2.09 (0.702–6.222) | 0.186 |  |  |  |
| No | 859 (97.0) | 66 (7.7) | Reference |  |  |  |  |
| Coronary heart disease, CHD | |  |  |  |  |  |  |
| Yes | 53 (6.0) | 3 (5.7) | 0.686 (0.208–2.258) | 0.535 |  |  |  |
| No | 833 (94.0) | 67 (8.0) | Reference |  |  |  |  |
| Cerebrovascular disease, CVD | |  |  |  |  |  |  |
| Yes | 17 (1.9) | 2 (11.8) | 1.571 (0.352–7.011) | 0.554 |  |  |  |
| No | 869 (98.1) | 68 (7.8) | Reference |  |  |  |  |
| Anemia |  |  |  |  |  |  |  |
| Yes | 24 (2.7) | 2 (8.3) | 1.061 (0.244–4.61) | 0.937 |  |  |  |
| No | 862 (97.3) | 68 (7.9) | Reference |  |  |  |  |
| Chronic obstructive pulmonary disease, COPD | | |  |  |  |  |  |
| Yes | 16 (1.8) | 2 (12.5) | 1.685 (0.375–7.567) | 0.496 |  |  |  |
| No | 870 (98.2) | 68 (7.8) | Reference |  |  |  |  |
| Tumor |  |  |  |  |  |  |  |
| Yes | 21 (2.4) | 1 (4.8) | 0.577 (0.076–4.363) | 0.594 |  |  |  |
| No | 865 (97.6) | 69 (8.0) | Reference |  |  |  |  |
| **Smoking** |  |  |  |  |  |  |  |
| Yes | 126 (14.2) | 7 (5.6) | 0.651 (0.291–1.455) | 0.295 |  |  |  |
| No | 760 (85.8) | 63 (8.3) | Reference |  |  |  |  |
| **Drinking** |  |  |  |  |  |  |  |
| Yes | 147 (16.6) | 13 (8.8) | 1.161 (0.618–2.18) | 0.643 |  |  |  |
| No | 739 (83.4) | 57 (7.7) | Reference |  |  |  |  |
| **Exposure to SFTSV patients** | |  |  |  |  |  |  |
| Yes | 22 (2.5) | 1 (4.6) | 0.549 (0.073–4.141) | 0.560 |  |  |  |
| No | 864 (97.5) | 69 (8.0) | Reference |  |  |  |  |
| **History of tick bite** |  |  |  |  |  |  |  |
| Yes | 176 (19.9) | 17 (9.7) | 1.325 (0.747–2.351) | 0.335 |  |  |  |
| No | 710 (80.1) | 53 (7.5) | Reference |  |  |  |  |
| **Farming frequency in last three months** | |  |  |  |  |  |  |
| ≥4/weeks | 396 (44.7) | 36 (9.1) | 1.341 (0.823–2.186) | 0.239 |  |  |  |
| <4/weeks | 490 (55.3) | 34 (6.9) | Reference |  |  |  |  |
| **Crops planting in last three months** | |  |  |  |  |  |  |
| Vegetables |  |  |  |  |  |  |  |
| Yes | 650 (73.4) | 56 (8.6) | 1.495 (0.816–2.739) | 0.193 |  |  |  |
| No | 236 (26.6) | 14 (5.9) | Reference |  |  |  |  |
| Rapeseed |  |  |  |  |  |  |  |
| Yes | 394 (44.5) | 37 (9.4) | 1.442 (0.884–2.351) | 0.143 |  |  |  |
| No | 492 (55.5) | 33 (6.7) | Reference |  |  |  |  |
| Tea |  |  |  |  |  |  |  |
| Yes | 136 (15.4) | 11 (8.1) | 1.031 (0.527–2.017) | 0.930 |  |  |  |
| No | 750 (84.6) | 59 (7.9) | Reference |  |  |  |  |
| **Forest working** |  |  |  |  |  |  |  |
| Yes | 60 (6.8) | 5 (8.3) | 1.064 (0.412–2.752) | 0.898 |  |  |  |
| No | 826 (93.2) | 65 (7.9) | Reference |  |  |  |  |
| **Close contact with domestic animals in recent two weeks** | | |  |  |  |  |  |
| Dogs |  |  |  |  |  |  |  |
| Yes | 288 (32.5) | 21 (7.3) | 0.881 (0.518–1.5) | 0.641 |  |  |  |
| No | 598 (67.5) | 49 (8.2) | Reference |  |  |  |  |
| Cats |  |  |  |  |  |  |  |
| Yes | 86 (9.7) | 16 (18.6) | 3.158 (1.717–5.807) | <0.001 |  | 2.648 (1.419–4.941) | 0.002 |
| No | 800 (90.3) | 54 (6.8) | Reference |  |  | Reference |  |
| Poultry |  |  |  |  |  |  |  |
| Yes | 254 (28.7) | 21 (8.3) | 1.072 (0.629–1.828) | 0.797 |  |  |  |
| No | 632 (71.3) | 49 (7.8) | Reference |  |  |  |  |
| Goats |  |  |  |  |  |  |  |
| Yes | 4 (0.4) | 1 (25.0) | 3.928 (0.403–38.263) | 0.239 |  |  |  |
| No | 882 (99.6) | 69 (7.8) | Reference |  |  |  |  |
| Cattle |  |  |  |  |  |  |  |
| Yes | 11 (1.2) | 2 (18.2) | 2.637 (0.559–12.451) | 0.221 |  |  |  |
| No | 875 (98.8) | 68 (7.8) | Reference |  |  |  |  |
| Pigs |  |  |  |  |  |  |  |
| Yes | 72 (8.1) | 8 (11.1) | 1.516 (0.696–3.305) | 0.295 |  |  |  |
| No | 814 (91.9) | 62 (7.6) | Reference |  |  |  |  |

The data were presented as *n* (proportion, %) until otherwise denoted. *OR*: odds ratio; *CI:* confidence interval; IQR: interquartile range.

Supplemental Table 9. The proportion of the IgG antibody titer in relate to NAb

| Titer of IgG antibody | NAb | | *P** |
| --- | --- | --- | --- |
|  | Positive | Negative |  |
| 1:80 | 15 (28.85) | 37 (71.15) | < 0.001 |
| 1:160 | 34 (53.97) | 29 (46.03) |  |
| 1:320 | 34 (79.07) | 9 (20.93) |  |
| 1:640 | 26 (83.87) | 5 (16.13) |  |
| 1:1280 | 5 (83.33) | 1 (16.67) |  |
| 1:2560 | 2 (100.00) | 0 (0.00) |  |

**P* value was calculated by chi-square trend test.

Supplemental Table 10. The risk factors for anti-SFTSV seropositive response of IgG antibody for different ages people by univariate analysis.

| Variables | < 60 years old (*n* = 458) | | |  | 60–70 years old (*n* = 189) | | |  | ≥ 70 years old (*n* = 239) | | |  |
| --- | --- | --- | --- | --- | --- | --- | --- | --- | --- | --- | --- | --- |
|  | Positive (rate, %) | *OR* (95% *CI*) | *P* |  | Positive (rate, %) | *OR* (95% *CI*) | *P* |  | Positive (rate, %) | *OR* (95% *CI*) | *P* |  |
| **Gender** |  |  |  |  |  |  |  |  |  |  |  |  |
| Female | 41(10.4) | 1.344(0.510, 3.541) | 0.550 |  | 22(17.9) | 3.376(1.111, 10.258) | 0.032 |  | 26(20.3) | 1.409(0.719, 2.761) | 0.317 |  |
| Male | 5(7.9) | Reference |  |  | 4(6.1) | Reference |  |  | 17(15.3) | Reference |  |  |
| **Underlying diseases** | | | |  |  |  |  |  |  |  |  |  |
| Hypertension | |  |  |  |  |  |  |  |  |  |  |  |
| Yes | 10(14.5) | 1.662(0.783, 3.529) | 0.186 |  | 11(17.7) | 1.610(0.691, 3.751) | 0.269 |  | 14(16.1) | 0.813(0.403, 1.639) | 0.563 |  |
| No | 36(9.25) | Reference |  |  | 15(11.8) | Reference |  |  | 29(19.1) | Reference |  |  |
| Diabetes |  |  |  |  |  |  |  |  |  |  |  |  |
| Yes | 1(11.1) | 1.122(0.137, 9.178) | 0.914 |  | 2(25.0) | 2.181(0.416, 11.433) | 0.356 |  | 2(20.0) | 1.146(0.235, 5.598) | 0.866 |  |
| No | 45(10.0) | Reference |  |  | 24(13.3) | Reference |  |  | 41(17.9) | Reference |  |  |
| Coronary heart disease, CHD | | |  |  |  |  |  |  |  |  |  |  |
| Yes | 0(0.0) | - | - |  | 3(12.0) | 0.836(0.231, 3.020) | 0.785 |  | 3(13.0) | 0.660(0.187, 2.329) | 0.518 |  |
| No | 46(10.2) |  |  |  | 23(14.0) | Reference |  |  | 40(18.5) | Reference |  |  |
| Cerebrovascular disease, CVD | |  |  |  |  |  |  |  |  |  |  |  |
| Yes | 0(0.0) | - | - |  | 1(16.7) | 1.264(0.142, 11.273) | 0.834 |  | 1(11.1) | 0.560(0.068, 4.595) | 0.589 |  |
| No | 46(10.1) |  |  |  | 25(13.7) | Reference |  |  | 42(18.3) | Reference |  |  |
| Anemia |  |  |  |  |  |  |  |  |  |  |  |  |
| Yes | 1(7.7) | 0.741(0.094, 5.830) | 0.776 |  | 0(0.0) | - | - |  | 0(0.0) | - | - |  |
| No | 45(10.1) | Reference |  |  | 26(14.4) |  |  |  | 43(18.2) |  |  |  |
| Chronic obstructive pulmonary disease, COPD | | |  |  |  |  |  |  |  |  |  |  |
| Yes | 0(0.0) | - | - |  | 0(0.0) | - | - |  | 4(36.4) | 2.769(0.773, 9.919) | 0.118 |  |
| No | 46(10.1) |  |  |  | 26(14.1) |  |  |  | 39(17.1) | Reference |  |  |
| Tumor |  |  |  |  |  |  |  |  |  |  |  |  |
| Yes | 2(15.4) | 1.657(0.356, 7.717) | 0.520 |  | 0(0.0) | - | - |  | 0(0.0) | - | - |  |
| No | 44(9.9) | Reference |  |  | 26(14.2) |  |  |  | 43(18.1) |  |  |  |
| **Smoking** |  |  |  |  |  |  |  |  |  |  |  |  |
| Yes | 1(3.3) | 0.293(0.039, 2.206) | 0.234 |  | 2(5.1) | 0.283(0.064, 1.257) | 0.097 |  | 10(17.5) | 0.961(0.440, 2.095) | 0.920 |  |
| No | 45(10.5) | Reference |  |  | 24(16.0) | Reference |  |  | 33(18.1) | Reference |  |  |
| **Drinking** |  |  |  |  |  |  |  |  |  |  |  |  |
| Yes | 5(9.3) | 0.903(0.341, 2.396) | 0.838 |  | 4(8.3) | 0.492(0.160, 1.507) | 0.214 |  | 11(24.4) | 1.638(0.751, 3.567) | 0.214 |  |
| No | 41(10.2) | Reference |  |  | 22(15.60) | Reference |  |  | 32(16.5) | Reference |  |  |
| **Exposure to SFTS patients** | |  |  |  |  |  |  |  |  |  |  |  |
| Yes | 0(0.0) | - | - |  | 0(0.0) | - | - |  | 2(33.3) | 2.341(0.415, 13.245) | 0.335 |  |
| No | 46(10.3) |  |  |  | 26(14.1) |  |  |  | 41(17.6) | Reference |  |  |
| **History of tick bite** |  |  |  |  |  |  |  |  |  |  |  |  |
| Yes | 15(17.9) | 2.405(1.232, 4.694) | 0.010 |  | 10(20.0) | 1.922(0.808, 4.573) | 0.140 |  | 7(16.7) | 0.894(0.368, 2.174) | 0.806 |  |
| No | 31(8.3) | Reference |  |  | 16(11.5) | Reference |  |  | 36(18.3) | Reference |  |  |
| **Farming frequency in last three months** | | |  |  |  |  |  |  |  |  |  |  |
| ≥4/weeks | 29(13.5) | 2.073(1.105, 3.890) | 0.023 |  | 19(19.0) | 2.748(01.095, 6.891) | 0.031 |  | 17(21.0) | 1.349(0.683, 2.663) | 0.389 |  |
| <4/weeks | 17(7.0) | Reference |  |  | 7(7.9) | Reference |  |  | 26(16.5) | Reference |  |  |
| **Crops planting in last three months** | | | |  |  |  |  |  |  |  |  |  |
| Vegetables |  |  |  |  |  |  |  |  |  |  |  |  |
| Yes | 35(12.1) | 1.979(0.977, 4.010) | 0.058 |  | 22(15.5) | 1.971(0.642, 6.046) | 0.235 |  | 32(20.3) | 1.616(0.767, 3.403) | 0.206 |  |
| No | 11(6.5) | Reference |  |  | 4(8.5) | Reference |  |  | 11(13.6) | Reference |  |  |
| Rapeseed |  |  |  |  |  |  |  |  |  |  |  |  |
| Yes | 14(15.2) | 1.873(0.954, 3.678) | 0.068 |  | 9(18.0) | 1.575(0.652, 3.806) | 0.313 |  | 8(19.1) | 1.089(0.464, 2.554) | 0.844 |  |
| No | 32(8.7) | Reference |  |  | 17(12.2) | Reference |  |  | 35(17.8) | Reference |  |  |
| Tea |  |  |  |  |  |  |  |  |  |  |  |  |
| Yes | 10(10.4) | 1.456(0.689, 3.078) | 0.325 |  | 9(27.3) | 3.066(1.226, 7.669) | 0.017 |  | 10(37.0) | 3.191(1.343, 7.577) | 0.009 |  |
| No | 36(9.4) | Reference |  |  | 17(10.9) | Reference |  |  | 33(15.6) | Reference |  |  |
| **Forest working** |  |  |  |  |  |  |  |  |  |  |  |  |
| Yes | 5(17.2) | 1.972(0.714, 5.445) | 0.190 |  | 1(6.7) | 0.425(0.054, 3.382) | 0.419 |  | 2(12.5) | 0.634(0.139, 2.899) | 0.557 |  |
| No | 41(9.6) | Reference |  |  | 25(14.4) | Reference |  |  | 41(18.4) | Reference |  |  |
| **Close contact with domestic animals in recent two weeks** | | |  |  |  |  |  |  |  |  |  |  |
| Dogs |  |  |  |  |  |  |  |  |  |  |  |  |
| Yes | 14(9.9) | 0.982(0.506, 1.903) | 0.957 |  | 9(13.4) | 0.958(0.402, 2.286) | 0.924 |  | 15(18.8) | 1.080(0.539, 2.161) | 0.829 |  |
| No | 32(10.1) | Reference |  |  | 17(13.9) | Reference |  |  | 28(17.6) | Reference |  |  |
| Cats |  |  |  |  |  |  |  |  |  |  |  |  |
| Yes | 4(15.4) | 1.688(0.555, 5.133) | 0.356 |  | 9(45.0) | 7.316(2.655, 20.157) | 0.000 |  | 10(25.0) | 1.677(0.748, 3.760) | 0.210 |  |
| No | 42(9.7) | Reference |  |  | 17(10.1) | Reference |  |  | 33(16.6) | Reference |  |  |
| Poultry |  |  |  |  |  |  |  |  |  |  |  |  |
| Yes | 15(12.6) | 1.433(0.744, 2.760) | 0.282 |  | 13(18.1) | 1.763(0.767, 4.053) | 0.182 |  | 13(20.6) | 1.265(0.612, 2.614) | 0.525 |  |
| No | 31(9.1) | Reference |  |  | 13(11.1) | Reference |  |  | 30(17.1) | 0.525 |  |  |
| Goats |  |  |  |  |  |  |  |  |  |  |  |  |
| Yes | 0(0.0) | - | - |  | 0(0.0) | - | - |  | 2(66.7) | 9.512(0.842, 107.40) | 0.069 |  |
| No | 46(10.1) |  |  |  | 26(13.8) |  |  |  | 41(17.4) | Reference |  |  |
| Cattle |  |  |  |  |  |  |  |  |  |  |  |  |
| Yes | 1(25.0) | 3.030(0.309, 29.739) | 0.342 |  | 1(33.3) | 3.22(0.281, 36.838) | 0.347 |  | 1(25.0) | 1.532(0.155, 15.090) | 0.715 |  |
| No | 45(9.9) | Reference |  |  | 25(13.4) | Reference |  |  | 42(17.9) | Reference |  |  |
| Pigs |  |  |  |  |  |  |  |  |  |  |  |  |
| Yes | 6(22.2) | 2.793(1.065, 7.322) | 0.037 |  | 7(23.3) | 2.243(0.848, 5.930) | 0.104 |  | 0(0.0) | - | - |  |
| No | 40(9.3) | Reference |  |  | 19(12.0) | Reference |  |  | 43(19.2) |  |  |  |

Note: *OR*: odds ratio; *CI*: confidence interval.


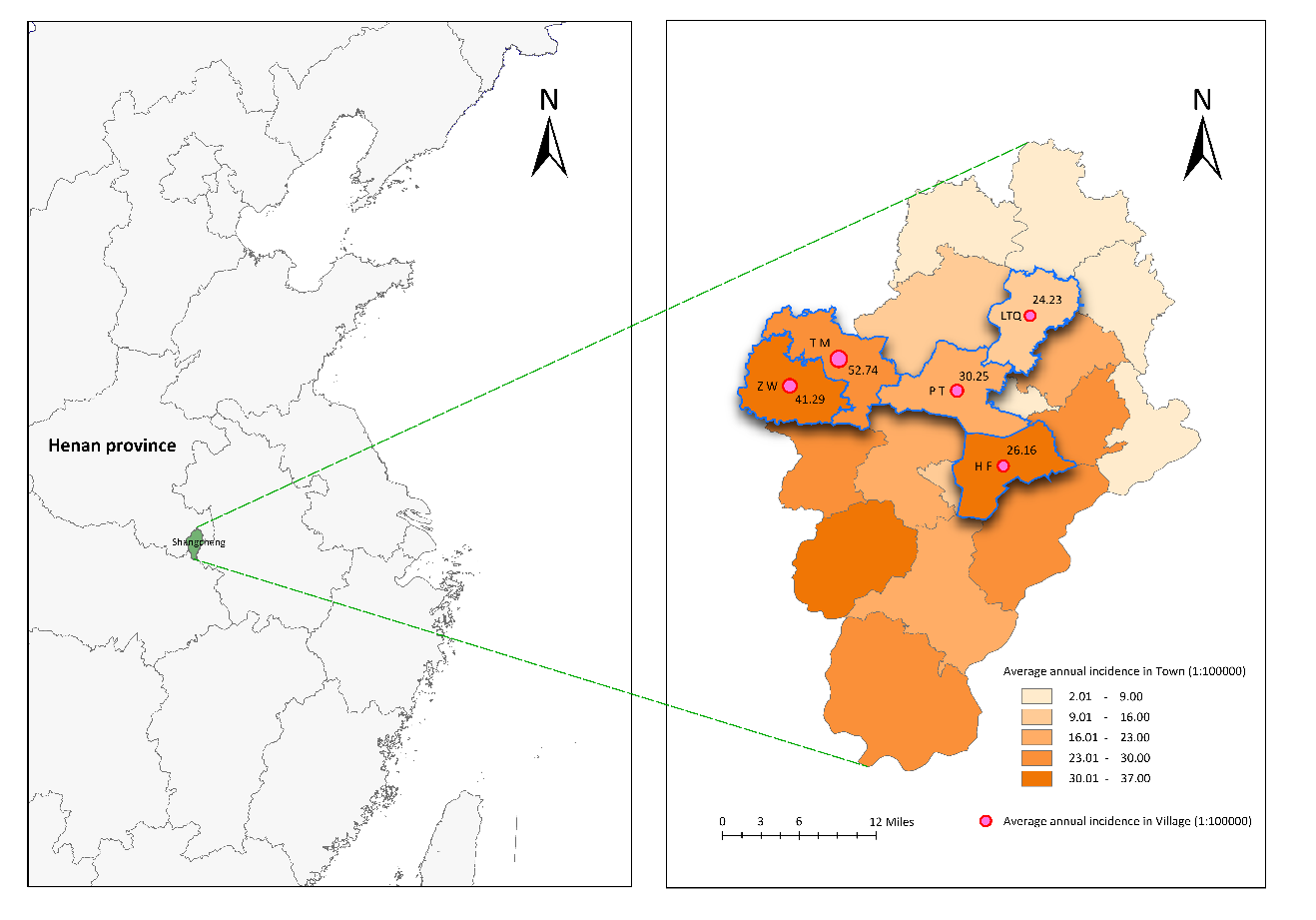
**Supplemental Figure 1. Location of the study sites, Shangcheng County in Henan Province, and the villages for sampling.**

The left panel marked the study site of Shangcheng County in Henan Province, the county with the highest SFTS incidence in China. The zoomed map marked the selected villages where the sampling was performed.

TM, Tumiao village in Wangqiao town; ZW, Zhaowan village in Wanggang town; PT, Pingtang village in Nianyushan town; HF, Hongfan village Guanmiao town; LTQ, Longtouqiao village in Hefengqiao town. The average annual incidence of each town was marked in red circle and number
